# The deubiquitinase USP38 affects cellular functions through interacting with LSD1

**DOI:** 10.1186/s40659-018-0201-8

**Published:** 2018-11-29

**Authors:** Wenbin Liu, Qi Zhang, Yuanyuan Fang, Yanan Wang

**Affiliations:** 1 Hubei Key Laboratory of Animal Nutrition and Feed Science, Wuhan Polytechnic University, Wuhan, Hubei China; 2 College of Health Sciences and Nursing, Wuhan Polytechnic University, No. 68 Southern Xuefu Road, Wuhan, 430023 Hubei China; 3 College of Life Sciences, Wuhan University, Wuhan, Hubei China

**Keywords:** LSD1, Deubiquitinase, USP38, Proliferation

## Abstract

**Background:**

Deubiquitination is a posttranslational protein modification prevalent in mammalian cells. Deubiquitinases regulate the functions of the target protein by removing its ubiquitin chain. In this study, the effects of the deubiquitinase USP38’s functions on the LSD1 protein and on cell physiology were investigated.

**Materials and methods:**

Western blotting, real-time quantitative PCR, immunoprecipitation, denaturing immunoprecipitation and luciferase reporter assays were used to analyze the protein stability, protein interactions and changes in the ubiquitin chain. Cell proliferation assays, colony formation assays, drug treatments and western blotting were used to explore the functions of USP38 in cells.

**Results:**

The deubiquitinase USP38 stabilizes protein LSD1 in cells by binding LSD1 and cleaving its ubiquitin chain to prevent the degradation of LSD1 by the intracellular proteasome. USP38 enhances the ability of LSD1 to activate signaling pathways and hence promotes cellular abilities of proliferation and colony formation through interacting with LSD1. Furthermore, USP38 enhances the drug tolerance of human colon cancer cells.

**Conclusions:**

USP38 is an LSD1-specific deubiquitinase that affects cellular physiology through interacting with LSD1.

## Background

Ubiquitin is a small peptide composed of 76 amino acids that exists in eukaryotic cells. Ubiquitin covalently attaches to protein via ligases and thus affects the stability, half-life, localization and function of the attached protein [1–4], consequently influencing cellular behaviors, such as proliferation, differentiation, migration, secretion and apoptosis. Ubiquitination is a biological process orchestrated by a cascade of enzymes including ubiquitin-activating enzymes (E1), ubiquitin-conjugating enzymes (E2), and ubiquitin ligases (E3) that mediate the transfer of one or more ubiquitin molecules onto target proteins [2, 4].

Ubiquitination is a posttranslational protein modification that differs from other protein modifications, such as phosphorylation, glycosylation, acetylation and neddylation, etc. All of these modifications are regulated precisely. Deubiquitination is a reverse process of ubiquitination that removes ubiquitin chains from the target proteins, thus affecting the fate and function of the target proteins, via a process performed by the deubiquitinases (DUBs). In the human genome, there are approximately 95 DUBs that belong to six families, ubiquitin C-terminal hydrolases (UCHs), ubiquitin specific processing proteases (USPs), Jab1/Pab1/MPN domain-containing metalloenzymes (JAMMs), Otu-domain ubiquitin aldehyde-binding proteins (OTUs), ataxin-3/Josephin proteins, and monocyte chemotactic protein induced proteases (MCPIPs). The USPs are the largest family and comprise more than 50 proteins with conserved domains and catalytic sites [4].

The alternative name of lysine-specific histone demethylase 1A (LSD1) is KDM1A. LSD1 is a histone demethylase that binds histone deacetylase 1 (HDAC1), REST corepressor 1 (CoRest) and PHD finger protein 21A (BHC80) to form a protein complex that regulates gene transcription and signaling pathways including the androgen receptor, signal transducer and activator of transcription 1 (STAT1), signal transducer and activator of transcription 3 (STAT3) and hypoxia-inducible factor 1 alpha (HIF-1α) pathways. The LSD1 protein level was reported to be changed and differentially regulated during different biological processes, such as myocyte and neuron differentiation [5–11]. Moreover, the dysregulation of LSD1 is involved in carcinogenesis. For example, LSD1 and estrogen-related receptor (ERRα) coregulate several target genes involved in cell migration and promote cancer cell invasion [12]. LSD1 depletion enhances tumor immunogenicity and T cell infiltration in poorly immunogenic tumors [13]. Furthermore, LSD1 is a potential regulator of ovarian cancer cell progression [14] and regulates autophagy via the mTOR signaling pathway in ovarian cancer cells [15]. LSD1 is also responsible for maintaining cancer stem cell self-renewal and tumorigenicity in hepatocellular carcinoma [16]. Therefore, LSD1 is an important molecule that should be precisely regulated, and the regular change in the LSD1 protein level indicates the regulation by the posttranslational ubiquitination and deubiquitination.

By searching a protein expression bank containing 80 human deubiquitinases, we identified the USP38 protein as a potential deubiquitinase of LSD1 in this study. USP38 is a member of the USP family and research on its functions is limited. However, USP38 was reported to be associated with the development of primary breast cancer [17]. In addition, USP38 expression is upregulated in the lung tissue in chronic obstructive pulmonary disease [18]. USP38 is also related to microbial infection caused by *Plasmodium falciparum* [19]. USP38 negatively regulates type I interferon (IFN) signaling by targeting the active form of TANK-binding kinase 1 (TBK1), a component of the type I IFN signaling pathway, for degradation [20].

This study revealed that USP38 is a deubiquitinase of LSD1 and affects cellular physiology by regulating the functions of LSD1.

## Methods

### Cells, antibodies and other reagents

The human embryonic kidney cell line HEK293T and the colon cancer cell line SW48 were cultured in Dulbecco’s Modified Eagle’s Medium (DMEM) and the colon cancer cell line HCT116 was cultured in McCoy’s 5A medium supplemented with 10% fetal bovine serum (FBS). Wild-type and LSD1 gene knockout HCT116 cell lines were supplied by the laboratory [21]. A cell counting kit 8 (CCK-8) was purchased from Dojindo Laboratories (Kumamoto Technology Research Park, Japan). Puromycin was purchased from Gene Operation (Ann Arbor, USA). MG 132 was from Selleckchem LLC (Houston, USA). Cycloheximide (CHX) and the mouse anti-Flag antibody (M2) were purchased from Sigma (Saint Louis, USA), and anti-GAPDH (glyceraldehyde-3-phosphate dehydrogenase) and anti-LSD1 antibodies were purchased from ABclonal Biotech Co (College Park, USA). Mouse anti-HA and anti-Myc antibodies were purchased from MBL International (Woburn, USA). ProteinA/G magnetic beads were purchased from Biotool Company (Shanghai, China). The USP38 expression plasmid pHAGE-6tag-Flag-USP38 and the signaling pathway luciferase assay plasmids were provided by Xiaodong Zhang, Wuhan University.

### Gene cloning and expression

The primers used for polymerase chain reaction (PCR) were synthesized by Beijing Tianyi-Huiyuan Biotechnology Co., Ltd. For LSD1 amplification, the forward primer was 5′-AGTTCAGAATTCATGGAGCAGAAACTCATCTCTGAAGAGGATCTGTTAT CTGGGAAGAAGGCGGCAG-3′, and the reverse primer was 5′-TCAACATCTAGATCACATGCTTGGGGACTGC-3′. For PHD finger protein 15 (JADE2) amplification, the forward primer was 5′-AGTTCAAAGCTTATGTACCCATACGATGTTCCAGATTACGCT GAAGAGAAGAGGCGAAAATAC-3′, and the reverse primer was 5′-ATCTAGTCTAGATTAGGAGGCCAGTACGCCCATGC-3′. The LSD1 PCR product was digested with *Eco*RI and *Xba*I, and the JADE2 PCR product was digested with *Hin*dIII and *Xba*I; these products were ligated into the pcDNA3.1 digested with the same enzymes to construct the recombinant plasmids.

### Protein stability assay

HEK293T cells were cultured in DMEM supplemented with 10% FBS in a 24-well plate and cotransfected with 0.5 μg of pcDNA3-Myc-LSD1, 0.5 μg of pcDNA3-HA-JADE2 and 0.5 μg of pHAGE-6tag-Flag-USP38. After 24 h, the culture medium was replaced, and after an additional 48 h, the cells were washed with phosphate buffered saline (PBS), and lysed with 1% sodium dodecyl sulfate (SDS) lysis buffer (50 mM Tris–HCl, 1% SDS and 10% glycerol; pH 6.8) to harvest total proteins. Protein sample of 50 μg was subjected to SDS-PAGE (polyacrylamide gel electrophoresis) and western blot analysis to detect the protein level of LSD1. In addition, pcDNA3-Myc-LSD1 and pHAGE-6tag-Flag-USP38 were cotransfected into HEK293T cells, and 48 h later, 40 μM of CHX was used to treat cells for 0, 1, 2, 4 and 8 h. The cells were lysed with 1% SDS lysis buffer and total proteins were subjected to SDS-PAGE. Western blot analysis was used to detect protein stability and change in the level of LSD1. The changes in the endogenous LSD1 protein were also detected by western blotting.

### Immunoprecipitation

HEK293T cells were cultured in six-well plates in DMEM and cotransfected with 1.5 μg of the pcDNA3-Myc-LSD1 plasmid and 1.5 μg of the pHAGE-6tag-Flag-USP38 plasmid. After 48 h, the HEK293T cells were washed twice with PBS on ice and lysed with radio immunoprecipitation assay (RIPA) lysis buffer [150 mM NaCl, 0.1% SDS, 0.5% sodium deoxycholate, 1% Nonidet P-40, 50 mM Tris, 10 μg/mL aprotinin, 10 μg/mL leupeptin, 10 μg/mL pepstatin, and 1 mM phenylmethanesulfonyl fluoride (PMSF); pH 8.0] for 10 min on ice. The cell lysate was ultrasonicated 5 times for 5 s each, followed by centrifugation at 12,000 rpm for 10 min at 4 °C. The pellet was discarded, the protein mixture was incubated with rotation for 2 h at 4 °C; 0.5 μg of anti-Myc antibody was added, and the mixture was then incubated with rotation for an additional 2 h at 4 °C. After the addition of 8 μL of ProteinA/G magnetic beads, rotation continued for 4 h. After centrifugation, the bead pellets were washed twice with RIPA lysis buffer for 2 min each and were harvested (by centrifugation at 3000 rpm and 4 °C for 1 min). Then the pellets were boiled with loading buffer and subjected to SDS-PAGE and western blot analysis.

### Denaturing immunoprecipitation

After transfection with the recombinant plasmids (pcDNA3-Myc-LSD1, pHAGE-6tag-Flag-USP38 and pcDNA3-HA-Ub)for 48 h, HEK293T cells were treated with 20 μM MG132 for 8 h. Then the cells were washed with PBS once before lysis with 600 μL of RIPA buffer (with 0.5% SDS) to harvest total proteins. The lysate was ultrasonicated 5 times for 5 min each and centrifuged at 12,000 rpm for 10 min at 4 °C. The pellet was discarded. The supernatant was mixed with 8 μL of ProteinA/G magnetic beads previously bound to 0.5 μg of anti-Myc antibody (mouse IgG was used as a control) and incubated with rotation at 4 °C for 2 h. Then the beads were centrifuged at 2,000 rpm for 2 min, washed twice with RIPA buffer, and boiled with loading buffer. After centrifugation, 10 μL of the supernatant was subjected to SDS-PAGE and western blot analysis, in which an anti-HA antibody was used to detect the ubiquitinated LSD1 proteins.

### RNA extraction, reverse transcription and real-time quantitative PCR

Total RNAs were extracted according to the manual of the Trizol kit. A standard reverse transcription protocol was used to obtain cDNAs, and real-time quantitative PCR was then performed with a Bio-Rad CFX connect real-time system to analyze the changes in mRNA level.

### Luciferase reporter assay

HEK293T cells were cultured in 24-well plates and transfected with 0.5 μg of pcDNA3.1 or pcDNA3-Myc-LSD1, 0.1 μg of pGL3-Luc-STAT1 and 10 ng of pRL-TK plasmids. After 48 h, the HEK293T cells were washed with PBS and lysed with 100 μL of LAG II and 10 μL of lysis buffer (1% Triton X-100, 10% glycerol, 0.3% Tris and 0.03% dithiothreitol (DTT); pH 7.8). The cell lysate was vortexed for 20 min. After the firefly luciferase activity was measured, 10 μL of SG solution was added into the above mixture, and the Renilla luciferase activity was measured using a GLOMAX 20/20 luminometer and GLOMAX SIS V1.10.0 software.

### Cell proliferation assay

Viruses of pHAGE-6tag and pHAGE-6tag-Flag-USP38 were packaged in HEK293T cells and used to infect HCT116 cells for 2 days. Then, 1 μg/mL puromycin was used to treat HCT116 cells for 3 continuous days to select cell lines stably expressing the USP38 protein. After the cells were counted, 1 × 10^3^ HCT116 cells were seeded into 96-well microplates and incubated at 37 °C for 4 days. Cell viability was assayed according to the manual of CCK-8 kit. In detail, 90 μL of DMEM supplemented with 10% FBS and 10 μL of CCK-8 solution were used to culture cells at 37 °C for an additional 1 h, and the culture’s absorbance of the culture at λ = 450 nm was measured using a microplate reader.

### Colony formation assay

After HCT116 LSD1 wild-type or LSD1 knockout (±) cells were infected with virus particles of pHAGE-6tag or pHAGE-6tag-Flag-USP38 and selected with 1 μg/mL puromycin, 500 cells were seeded in six-well plate and incubated for ten (for LSD1 wild-type HCT116 cells) or 13 continuous days (for LSD1 knockout (±) HCT116 cells). Then, the cell colonies were stained with crystal violet, washed with water and dried. Colonies with more than 100 cells were counted.

### Drug tolerance assay

HCT116 stably expressing the USP38 protein and control HCT116 cells were counted, and 1 × 10^3^ cells were seeded in 96-well microplates. The cells were incubated at 37 °C for 5 h, after which the culture medium was replaced with fresh medium containing 40 ng/mL paclitaxel (PTX) or 12.5 µg/mL 5-fluorouracil (5-FU). The HCT116 cells were cultured with PTX or 5-FU for an additional 4 days. Cell viability was assessed with CCK-8 kit by incubating the cells in 90 μL of DMEM containing 10% FBS and 10 μL of CCK-8 solution at 37 °C for an additional 1 h. Subsequently, the absorbance was analyzed using a microplate reader at a wavelength of 450 nm.

### Statistical analysis

Statistical significance for the comparison of means was determined with Student’s *t*-test and defined as *p* < 0.05. The data are presented as the means ± standard deviations (SDs).

## Results

### The deubiquitinase USP38 stabilizes the protein level of LSD1

**Fig. 1:**
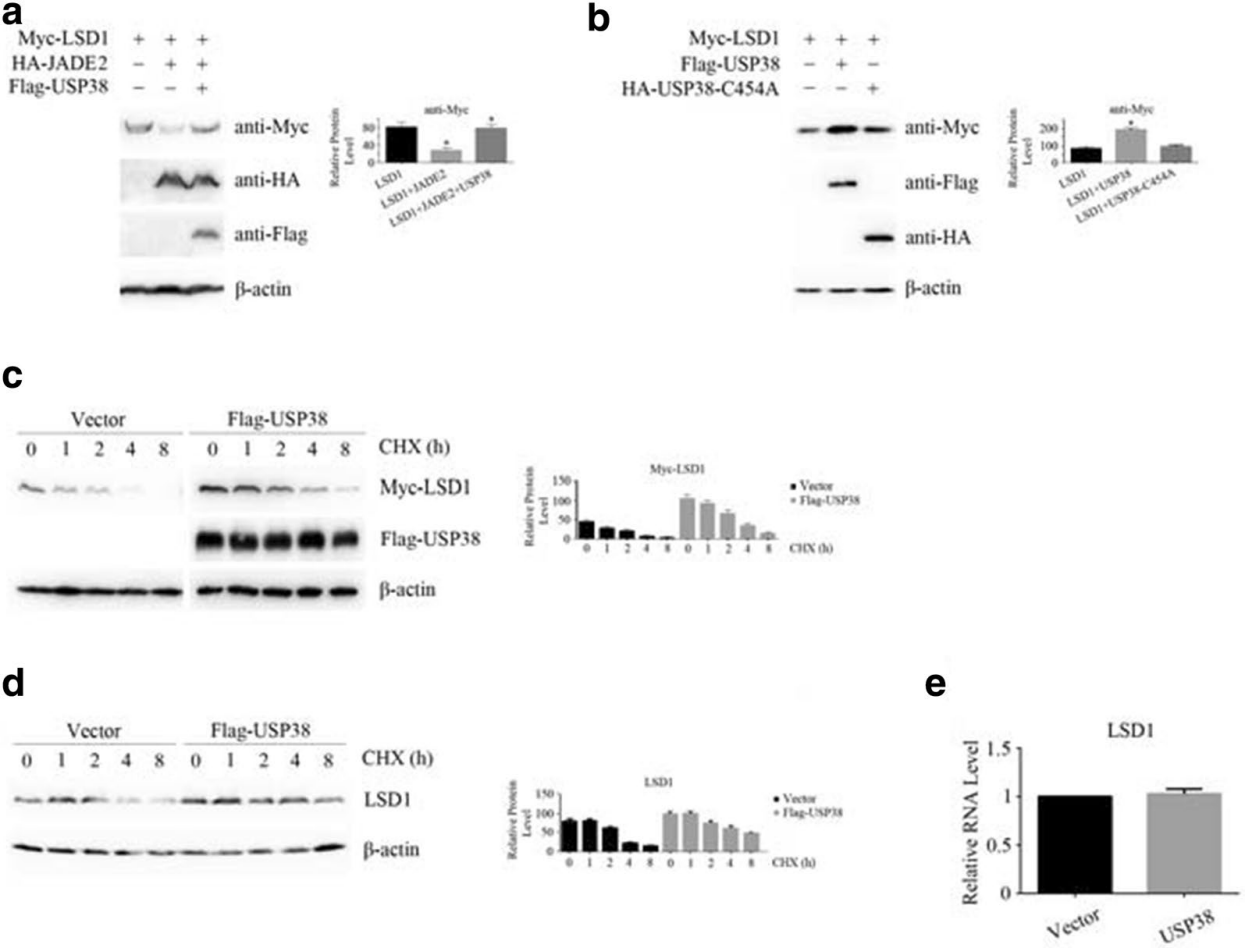
The LSD1 protein is stabilized by the deubiquitinase USP38. **a** After tagged proteins of LSD1, JADE2 and USP38 proteins were expressed in HEK293T cells, the change in the LSD1 protein level was measured by western blotting. An asterisk (*) indicates *p* < 0.05, compared to LSD1-expressing or LSD1 and JADE2-expressing cells. **b** After LSD1 and USP38 or the USP38-C454A mutant were expressed in HEK293T cells, the change in the LSD1 protein level was measured by western blotting. An asterisk (*) indicates *p* < 0.05, compared to LSD1-expressing cells. **c** The Myc-tagged LSD1 gene was transfected into HEK293T cells together with the vector or Flag-tagged USP38 gene, and the cells were treated with cycloheximide (CHX) for 8 h. Western blotting was then performed to detect the changes in the protein level of Myc-LSD1. **d** The vector or Flag-tagged USP38 gene was transfected into HEK293T cells and the cells were treated with CHX for 8 h. Western blotting was then performed to detect the changes in the protein level of endogenously expressed LSD1. **e** Total RNAs were extracted from the vector- and Flag-USP38-transfected HEK293T cells and real-time quantitative PCR was performed to detect the changes in the LSD1 mRNA level. Three independent experiments were performed

LSD1 ubiquitination requires the consecutive action of three enzymes: E1 ubiquitin-activating enzymes, E2 ubiquitin-conjugating enzymes, and E3 ubiquitin ligases. JADE2 is an important E2 of LSD1 and is involved in the ubiquitination and degradation of LSD1 [22]. The deubiquitination of human proteins involves approximately 95 deubiquitinases and similar peptides that regulate the functions of these proteins. We screened an 80-gene deubiquitinase bank and identified the USP38 protein as a potential deubiquitinase of LSD1. As shown in Fig. 1a, LSD1 protein level decreased when JADE2 was overexpressed in HEK293T cells. However, when JADE2 and USP38 were coexpressed together, the protein levels of exogenously expressed LSD1 did not change significantly (Fig. 1a), indicating that USP38 abolished the increase in the degradation of LSD1 by JADE2 and stabilized the intracellular LSD1 protein level. A cysteine (C) residue is located at position 454 in the USP domain of USP38 protein; this residue is the key cysteine residue for the catalytic activity of USP38 in removing the ubiquitin chain from the target protein. We mutated this cysteine (C) to an alanine (A) to construct a USP38 mutant (USP38-C454A), and we coexpressed this mutant with LSD1 in HCT116 cells. With the expression of USP38-C454A, the protein level of LSD1 did not change significantly, as shown in Fig. 1b. To further examine this question, we used the cell protein translation process inhibitor CHX to stop the synthesis of new cellular proteins. After treatment with 40 μM CHX for 8 h, in control cells (transfected with vector), the protein levels of total exogenously and endogenously expressed LSD1 were low and significantly decreased with time, as shown in Fig. 1c, d, while in USP38-expressing cells, the protein levels of LSD1 did not change significantly. After treatment with CHX, the half-life of exogenously expressed LSD1 was 1.7 h in vector-transfected cells, and 3.2 h in USP38-expressing cells. Furthermore, the half-life of endogenously expressed LSD1 protein was 2.7 h in vector-transfected cells and 7.9 h in USP38-expressing cells. These results indicate that USP38 can stabilize the LSD1 protein in cells. To determine whether the LSD1 protein level was increased by posttranslational modification or by gene transcription, we isolated total RNAs from vector- or USP38-transfected cells, and reverse transcription was performed to obtain cDNAs. The results of the real-time quantitative PCR analysis indicate that the mRNA levels of LSD1 in vector- or USP38-transfected cells were not significantly different (Fig. 1e). The above results imply that the protein level of LSD1 is regulated by JADE2 and USP38 and that this regulation occurs via posttranslational modification, not via gene transcription. USP38 does not activate LSD1 gene expression to promote the increase in the LSD1 protein level; instead, USP38 possibly deubiquitinates LSD1 and inhibits LSD1 degradation by the 26S proteasome, thus maintaining the LSD1 protein level in cell.

### The deubiquitinase USP38 binds LSD1

**Fig. 2:**
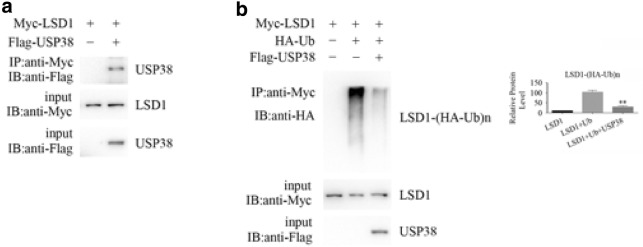
USP38 binds LSD1 and removes its ubiquitin chain. **a** Coimmunoprecipitation was performed to detect the interaction between LSD1 and USP38. **b** Denaturing immunoprecipitation was applied to confirm that USP38 removed the ubiquitin chain from the LSD1 protein specifically. Double asterisks (**) indicate *p* < 0.01, compared to LSD1- and ubiquitin-expressing cells. Three independent experiments were performed

To remove the ubiquitin chain from the target protein, the deubiquitinase should bind the target protein specifically and cleave the polyubiquitin chain from the protein. To investigate whether USP38 interacts with LSD1, we coexpressed Myc-LSD1 and Flag-USP38 in HEK293T cells. After immunoprecipitation of LSD1, we detected the associated USP38 (Fig. 2a); similarly, upon immunoprecipitation of USP38, we detected the associated LSD1 (data not shown). This result means the interaction between LSD1 and USP38 is possibly specific. By interacting with LSD1, USP38 may remove the ubiquitin chain from the protein LSD1 and thus maintain the LSD1 protein level in cells.

### The deubiquitinase USP38 removes the ubiquitin chain from the LSD1 protein

The deubiquitination of LSD1 requires USP38 to bind it and cleave its ubiquitin chain from it. To show that this process occurs, we performed denaturing immunoprecipitation. As shown in Fig. 2b, after the expression of HA-ubiquitin (HA-Ub) and treatment with the 26S proteasome inhibitor MG132, the ubiquitination level of LSD1 was increased, indicating that the exogenously expressed HA-ubiquitin was incorporated into the ubiquitin chain on LSD1 and was not degraded in a timely manner by the 26S proteasome because of the inhibitor MG132. Furthermore, HA-Ub was detected by the anti-HA antibody against the HA tag on the ubiquitin.

When USP38 was expressed in HEK293T cells, the ubiquitination level of LSD1 deceased compared to that in vector-transfected cells. This result indicates that USP38 removed or cleaved the ubiquitin chain (including HA-tagged ubiquitin, HA-Ub) from LSD1 and that these LSD1 proteins were not detected by the anti-HA antibody against HA-Ub (Fig. 2b). The IgG control did not produce a band in any lane (data not shown).

### Generation of the LSD1 single-allele knockout HCT116 cell line

**Fig. 3:**
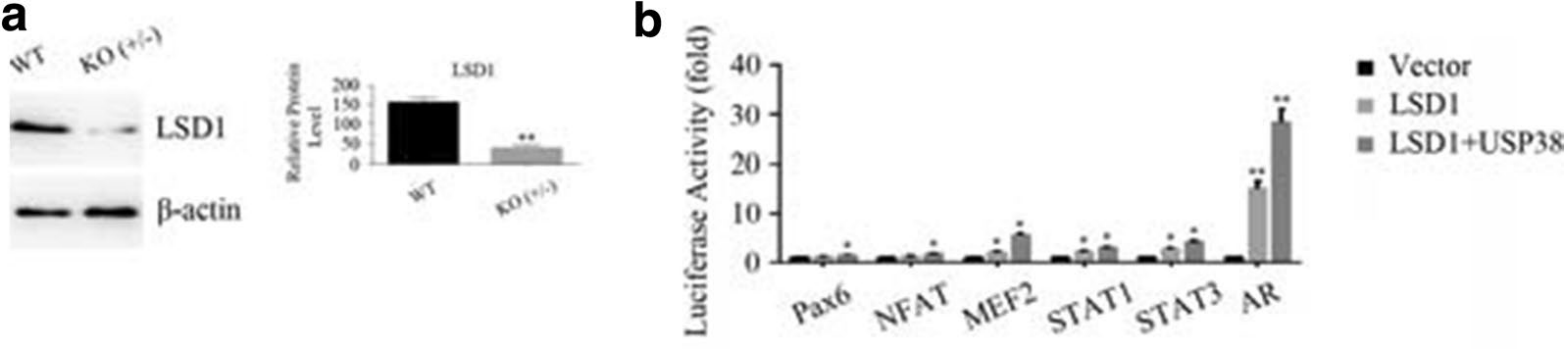
USP38 enhances the activation of signaling pathways activated by LSD1. **a** The protein levels of LSD1 in wild-type (WT, +/+) and single-allele knockout (KO, +/−) HCT116 cells. Double asterisks (**) indicate *p* < 0.01, compared to wild-type cells. **b** After the Myc-tagged LSD1 gene was transfected into HEK293T cells, luciferase assays were performed to detect the activation of signaling pathways. In addition, after Myc-tagged LSD1 and/or Flag-tagged USP38 genes were transfected into HEK293T cells, luciferase assays were performed to detect the activation of signaling pathways as well. An asterisk (*) indicates *p* < 0.05 and double asterisks (**) indicate *p* < 0.01, compared to vector-transfected cells. Three independent experiments were performed

In order to knock out LSD1 gene from the LSD1 wild-type HCT116 cell line (WT, +/+), we tried to perform somatic cell gene targeting based on the pAAV-LoxP-Neo and Cas9/CRISPR (clustered regularly interspaced short palindromic repeats) techniques but failed to obtain the LSD1 double-allele knockout HCT116 cell line. Only the LSD1 single-allele knockout HCT116 cell line (KO, +/−) was obtained, in which the expression of the LSD1 protein was much lower than that in LSD1 wild-type cell (Fig. 3a). It is possible that the two LSD1 alleles could not be completely knocked out or that the LSD1 double-allele knockout HCT116 cell line (KO, −/−) could not survive in DMEM or McCoy’s 5A medium supplemented with 10% FBS.

### The deubiquitinase USP38 enhances the activity of the signaling pathways activated by LSD1

LSD1 is a major demethylase for histone H3K4me2, and it regulates gene transcription and activates several signaling pathways, such as the STAT1 and androgen receptor signaling pathways [10, 23, 24]. To investigate the consequence of the removal of the ubiquitin chain from the LSD1 protein by USP38, we constructed a recombinant plasmid bank containing 45 reporter genes and cotransfected these plasmids with LSD1-expressing or USP38-expressing plasmid into HEK293T cells. As shown in Fig. 3b, after the overexpression of LSD1, the luciferase activities in certain signaling pathways, including androgen receptor (AR), STAT1, myocyte-specific enhancer factor 2 (MEF2), Pax6, STAT3 and nuclear factor of activated T-cells (NFAT) signaling pathways, were elevated significantly. This result means that these signaling pathways were activated. When LSD1-expressing and USP38-expressing plasmids were cotransfected, these signaling pathways were activated further (Fig. 3b). Luciferase activity was also measured in the LSD1 gene knock-out (KO, +/−) HCT116 cell lines. We did not detect a significant change in the luciferase activity when the USP38 protein was overexpressed (data not shown).

### The ubiquitinase USP38 promotes cell proliferation

**Fig. 4:**
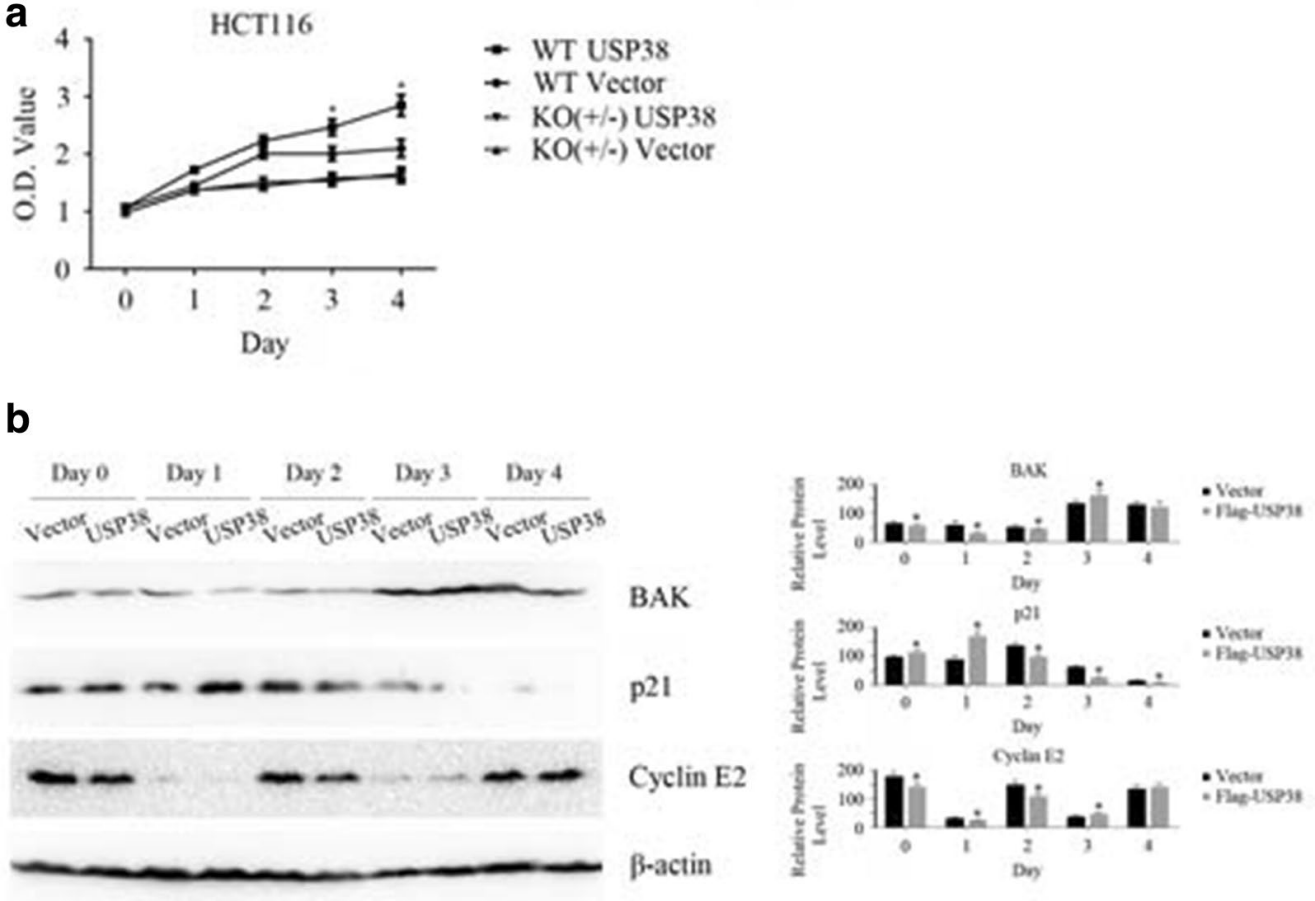
USP38 promotes the proliferation of HCT116 cells. **a** After the vector or Flag-tagged USP38 gene was transfected into LSD1 wild-type (WT, +/+) or single-allele knockout (KO, +/−) HCT116 cells, cell proliferation assays were performed with CCK-8 kits. An asterisk (*) indicates *p* < 0.05, compared to vector-transfected HCT116 wild-type cells. **b** After the vector or Flag-tagged USP38 gene was transfected into LSD1 wild-type (WT, +/+) HCT116 cells, western blotting was performed to detect the expression of cell cycle and apoptosis related proteins. An asterisk (*) indicates *p* < 0.05, compared to vector-transfected cells. Three independent experiments were performed

The results of the luciferase reporter assays indicate that the deubiquitinase USP38 may affect several signaling pathways and affect cell proliferation through the LSD1 protein. Endogenous LSD1 expression was observed in the HCT116 cell line. Thus, cell proliferation assays were performed to investigate the functions of LSD1 and USP38. As shown in Fig. 4a, in LSD1 wild-type HCT116 cells (WT, +/+), the overexpression of the LSD1 deubiquitinase USP38 promoted cell proliferation. However, when one LSD1 allele was knocked out and the intracellular LSD1 protein level was appreciably decreased, the overexpression of USP38 did not affect the proliferation of HCT116 cells (Fig. 4a). Thus, the LSD1 gene is very important for the function of USP38 in cell proliferation and USP38 promotes cell growth by interacting with the LSD1 protein, which regulates the function of USP38. After the expression of USP38 in LSD1 gene wild-type HCT116 cells, the protein levels of Bcl-2 homologous antagonist/killer (BAK), p21 and cyclin E2 decreased with individual patterns (Fig. 4b), while the protein levels of Bcl-2-associated agonist of cell death (BAD), Bcl-2 interacting mediator of cell death (BIM), Bcl-Xl, cyclin B1 and checkpoint kinase 2 (CHEK2) did not change (data not shown). If USP38 was overexpressed in LSD1 knockout HCT116 cells (KO, +/−, in which the LSD1 protein was downregulated), the protein levels of the above cell proliferation or apoptosis related proteins did not significantly change (data not shown). The above results indicate that USP38 affects the growth of cancer cells through interacting with LSD1 and changing the levels of other cell cycle or apoptosis related proteins.

### The deubiquitinase USP38 promotes cell colony formation ability

**Fig. 5:**
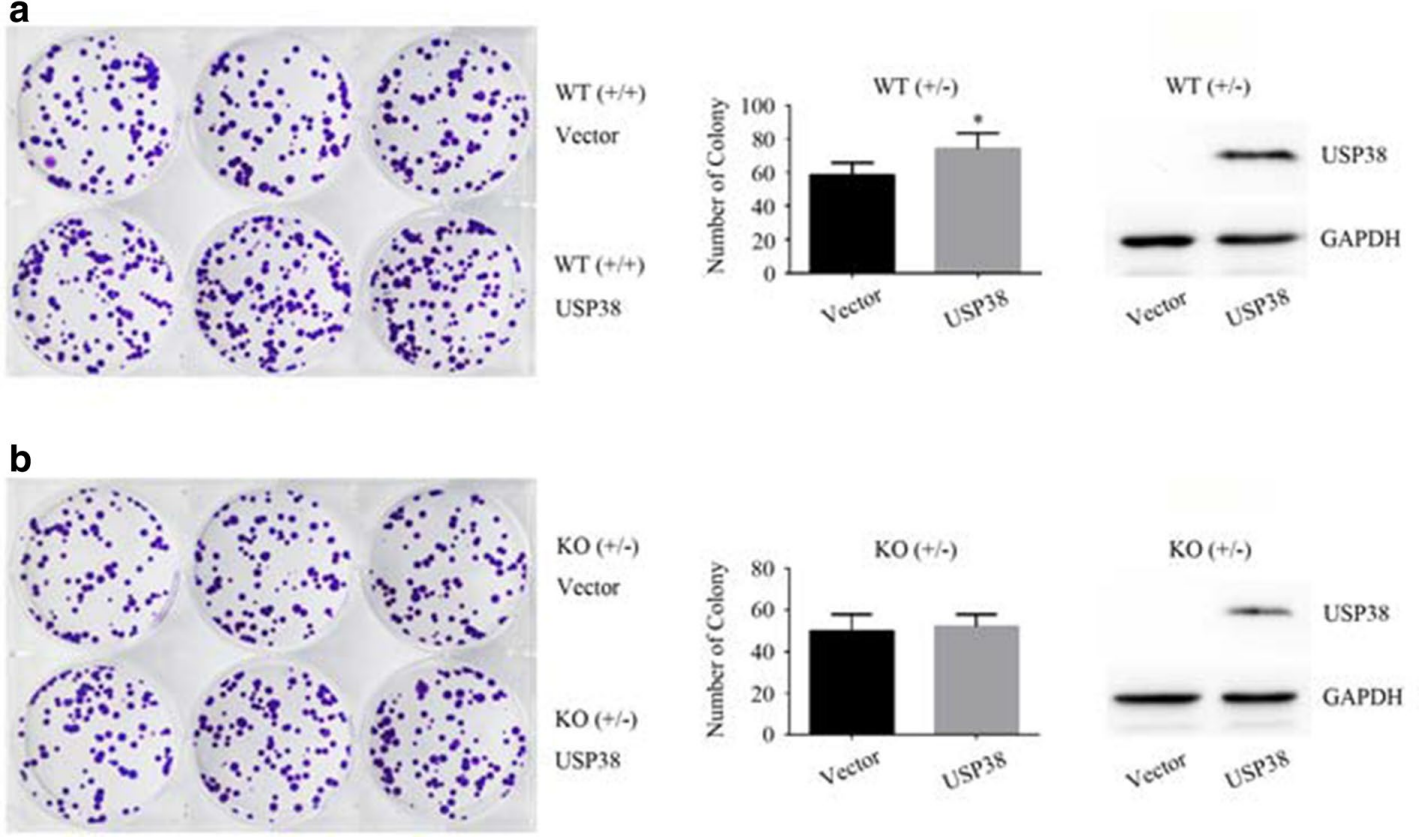
Colony formation analysis. **a** LSD1 wild-type HCT116 cells (WT, +/+) were transfected with packaged lentiviruses of pHAGE-6tag or pHAGE-6tag-Flag-USP38, the stable USP38-expressing cell lines were screened with puromycin, and a colony formation assay was performed for 10 days. An asterisk (*) indicates *p* < 0.05, compared to vector-transfected cells. **b** LSD1 single-allele knockout (KO, +/−) HCT116 cells were transfected with packaged lentiviruses of pHAGE-6tag or pHAGE-6tag-Flag-USP38 and screened with puromycin, a colony formation assay was then performed for 13 days. Three independent experiments were performed

Because USP38 promoted the proliferation of cancer cells, we wanted to know whether it promotes the colony formation ability of cancer cells, which is related to cell proliferation and carcinogenesis. As shown in Fig. 5a, the introduction of the USP38 protein promoted the colony formation ability of LSD1 wild-type HCT116 cells (WT, +/+). After one allele of the LSD1 gene was knocked-out (KO, +/−), the protein level of LSD1 declined noticeably (Fig. 3a), and USP38 did not appreciably promote the colony formation ability of HCT116 cells (Fig. 5b). Thus, USP38 promotes the colony formation ability of HCT116 cells through the interaction between USP38 and LSD1.

### The deubiquitinase USP38 promotes cellular drug tolerance

**Fig. 6:**
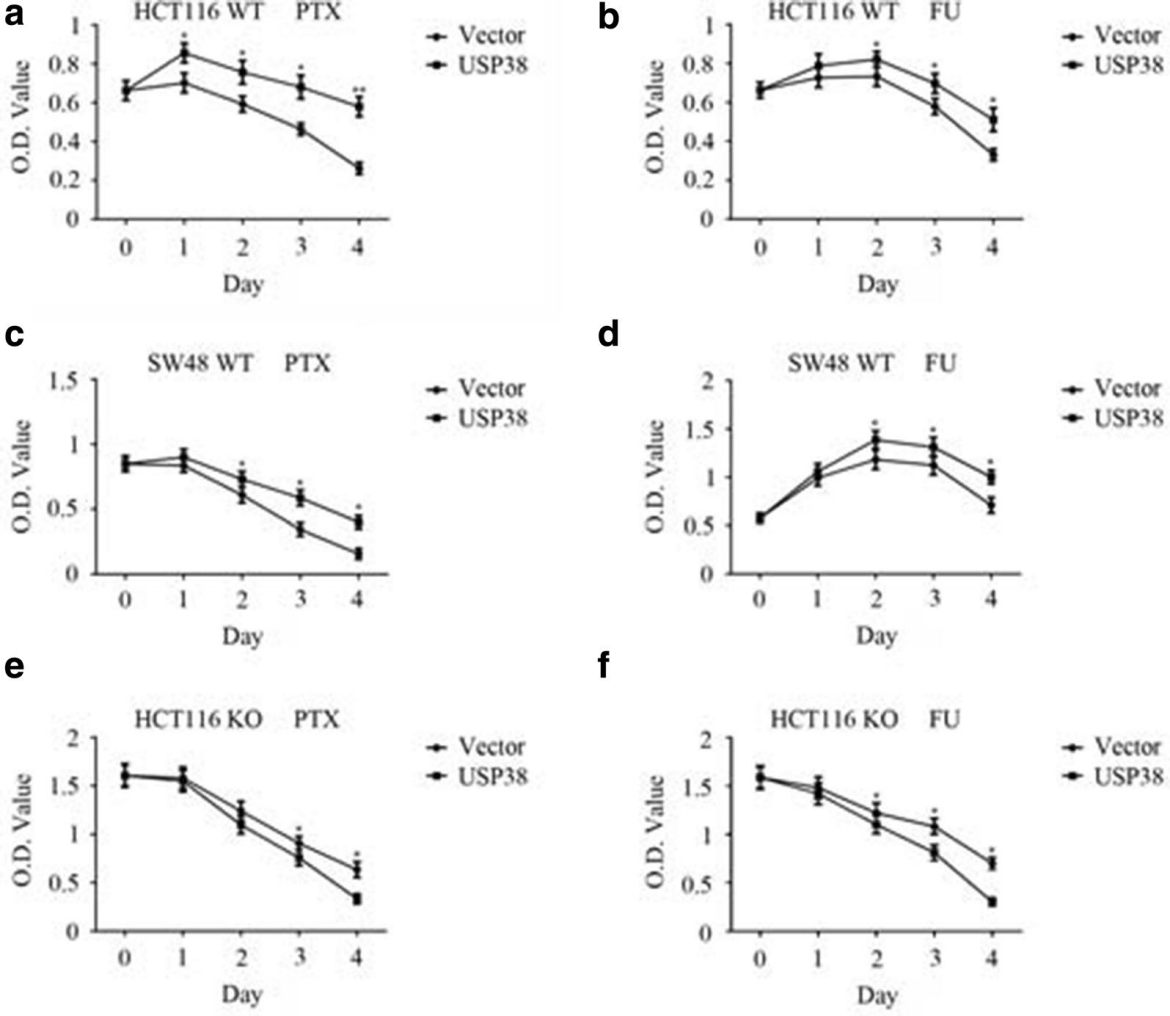
Drug tolerance assay. **a** HCT116 cells (LSD1 WT, +/+) stably expressing the USP38 protein and vector-transfected HCT116 cells (LSD1 WT, +/+) were treated with 40 ng/mL paclitaxel (PTX) for 4 days and CCK-8 was applied to measure cell proliferation. An asterisk (*) indicates *p* < 0.05 and double asterisks (**) indicate *p* < 0.01, compared to vector-transfected cells. **b** HCT116 cells (LSD1 WT, +/+) stably expressing the USP38 protein and vector-transfected HCT116 cells (LSD1 WT, +/+) were treated with 12.5 µg/mL 5-fluorouracil (5-FU) for 4 days and CCK-8 was applied to measure cell proliferation. An asterisk (*) indicates *p* < 0.05, compared to vector-transfected cells. **c** USP38 was expressed in SW48 cells (LSD1 WT, +/+), the cells were treated with PTX, and cell proliferation was analyzed. An asterisk (*) indicates *p* < 0.05, compared to vector-transfected cells. **d** USP38 was expressed in SW48 cells (LSD1 WT, +/+), the cells were treated with 5-FU, and cell proliferation was analyzed. An asterisk (*) indicates *p* < 0.05, compared to vector-transfected cells. **e** LSD1 single-allele knockout (KO, +/−) HCT116 cells were applied to be treated with PTX and cell proliferation was analyzed. An asterisk (*) indicates *p* < 0.05, compared to vector-transfected cells. **f** LSD1 single-allele knockout (KO, +/−) HCT116 cells were applied to be treated with 5-FU and cell proliferation was analyzed. An asterisk (*) indicates *p* < 0.05, compared to vector-transfected cells. Three independent experiments were performed

We checked the proliferation of cells under stress, namely, drug exposure. As shown in Fig. 6a, b, when LSD1 wild type HCT116 cells (WT, +/+) were treated with 40 ng/mL PTX or 12.5 µg/mL 5-FU, USP38 overexpression promoted HCT116 cell proliferation. Similar results were observed for drug treatment of another colon cancer cell line SW48 (Fig. 6c, d). When one allele of LSD1 was knocked out and the protein level of LSD1 was low, the proliferation of cells under stress was not promoted, but inhibited (Fig. 6e, f). Thus, by interacting with the LSD1 protein, USP38 helps cells resist stress imposed by drug treatment.

In conclusion, USP38 is a specific deubiquitinase of LSD1 and affects the functions of LSD1.

## Discussion

The posttranslational protein modifications of ubiquitination and deubiquitination regulate the levels of many proteins. One protein can be regulated by several deubiquitinases and one deubiquitinase can regulate several target proteins in cells. For example, the cell cycle-related protein p53 can bind USP4, USP7, USP10 and USP42, and then be deubiquitinated by these deubiquitinases [2, 25–27]. It was reported that USP28 is a deubiquitinase of LSD1 [28]. We hypothesize that other deubiquitinases also target LSD1 and regulate its functions in order for LSD1 to regulate the functions of other proteins more precisely. Through screening, we found that USP38 is a deubiquitinases of LSD1. Further studies are needed to explore whether USP28 and USP38 regulate LSD1 protein levels independently or act together. USP38 has been reported to regulate the functions of cell division cycle 25 (CDC25) and suppressor of defective silencing 3 protein homolog (SUDS3), so the relationship between USP38 and LSD1 is not one-to-one specific [29, 30].

By binding, USP38 cleaves the ubiquitin chain from the LSD1 protein and prevents it from degradation by the 26S proteasome, thus maintaining a certain LSD1 protein level in cells. However, we do not know whether this interaction is direct or indirect, because we could not induce the prokaryote *Escherichia coli* to express the fusion protein GST-USP38. The molecular weight of USP38 is 116 kDa, making the molecular weight of the fusion protein GST-USP38 larger, approximately 137 kDa, and thus it is very difficult for bacteria to express GST-USP38 ectopically. Therefore, we could not perform pull-down test to prove the direct interaction between USP38 and LSD1. When LSD1 is overexpressed in cells, it activates signaling pathways such as the STAT1, AR and STAT3 pathways. Because of USP38, the degradation of LSD1 is inhibited and its protein level is maintained, hence enhancing the activation of LSD1 target signaling pathways. Consequently, the activation of signaling pathways will modify cell behaviors, such as proliferation, differentiation and apoptosis, and resulting in body development or diseases.

By searching the Oncomime microarray database, we found that compared to its expression in normal tissue, USP38 is overexpressed in cervical cancer tissue (2.485-fold). Thus, consistent with our data on cell proliferation and colony formation, the deubiquitinase USP38 may promote carcinogenesis. Furthermore, the LSD1 protein was previously reported to be overexpressed in some carcinomas as well [31, 32].

## Conclusions

This study provides a deeper understanding of the complex functions and precise regulation of LSD1 and helps us to further understand the molecular mechanisms of body development and diseases.

Our data indicate that USP38 stabilizes the protein level of LSD1 in cells by binding and removing the ubiquitin chain from the LSD1 protein, and enhances LSD1-mediated activation of signaling pathways. Therefore, USP38 is a deubiquitinase of LSD1 and regulates its functions in the human embryonic kidney cell line HEK293T and the colon cancer cell lines HCT116 and SW48.

## Abbreviations

DUB: deubiquitinase
LSD1: lysine-specific histone demethylase 1A
UCH: ubiquitin C-terminal hydrolase
USP: ubiquitin specific processing protease
JAMM: Jab1/Pab1/MPN domain-containing metalloenzyme
OUT: out-domain ubiquitin aldehyde-binding protein
MCPIP: monocyte chemotactic protein induced protease
HDAC1: histone deacetylase 1
CoRest: REST corepressor
BHC80: PHD finger protein 21 A
STAT1: signal transducer and activator of transcription 1
STAT3: signal transducer and activator of transcription 3
HIF-1α: hypoxia-inducible factor 1 alpha
ERRα: estrogen-related receptor alpha
IFN: interferon
TBK1: TANK-binding kinase 1
DMEM: Dulbecco’s Modified Eagle’s Medium
FBS: fetal bovine serum
CCK-8: cell counting kit 8
CHX: cycloheximide
GAPDH: glyceraldehyde-3-phosphate dehydrogenase
PCR: polymerase chain reaction
JADE2: PHD finger protein 15
SD: standard deviation
PBS: phosphate buffered saline
SDS: sodium dodecyl sulfate
PAGE: polyacrylamide gel electrophoresis
RIPA: radio immunoprecipitation assay
DTT: dithiothreitol
PTX: paclitaxel
5-FU: 5-fluorouracil
CRISPR: clustered regularly interspaced short palindromic repeats
AR: androgen receptor
MEF2: myocyte-specific enhancer factor 2
NFAT: nuclear factor of activated T-cells
BAK: Bcl-2 homologous antagonist/killer
BAD: Bcl-2-associated agonist of cell death
BIM: Bcl-2 interacting mediator of cell death
CHEK2: checkpoint kinase 2
CDC25: cell division cycle 25
SUDS3: suppressor of defective silencing 3 protein homolog
